# Early Diagnosis, Treatment and Follow-Up of Cystic Echinococcosis in Remote Rural Areas in Patagonia: Impact of Ultrasound Training of Non-Specialists

**DOI:** 10.1371/journal.pntd.0001444

**Published:** 2012-01-10

**Authors:** Mario Del Carpio, Carlos Hugo Mercapide, Juan Carlos Salvitti, Leonardo Uchiumi, José Sustercic, Hector Panomarenko, Jorge Moguilensky, Eduardo Herrero, Gabriel Talmon, Marcela Volpe, Daniel Araya, Guillermo Mujica, Arnoldo Calabro, Sergio Mancini, Carlos Chiosso, Jose Luis Labanchi, Ricardo Saad, Sam Goblirsch, Enrico Brunetti, Edmundo Larrieu

**Affiliations:** 1 Rogelio Cortizo Hospital, Ingeniero Jacobacci, Río Negro Province, Argentina; 2 Artémides Zatti Hospital, Viedma, Río Negro Province, Argentina; 3 Ramón Carrillo Hospital, Bariloche, Rio Negro Province, Argentina; 4 Francisco López Lima Hospital, General Roca, Río Negro Province, Argentina; 5 El Bolsón Hospital, El Bolsón, Río Negro Province, Argentina; 6 Ministry of Health, Viedma, Río Negro Province, Argentina; 7 “Prozome” Laboratory Viedma, Viedma, Río Negro Province, Argentina; 8 Department of Medicine, University of Minnesota, Minneapolis, Minnesota, United States of America; 9 Division of Infectious and Tropical Diseases, University of Pavia, IRCCS S. Matteo Hospital Foundation, and WHO Collaborating Centre for Clinical Management of Cystic Echinococcosis, Pavia, Italy; 10 Ministry of Health, Viedma, Río Negro Province, Argentina; 11 Veterinary Faculty, National University of La Pampa, General Pico, La Pampa Province, Argentina; Universidad Nacional Autónoma de México, Mexico

## Abstract

Cystic echinococcosis (CE) is a chronic, complex and neglected disease caused by the larval stage of *Echinococcus granulosus*. The effects of this neglect have a stronger impact in remote rural areas whose inhabitants have no chances of being diagnosed and treated properly without leaving their jobs and travelling long distances, sometimes taking days to reach the closest referral center.

**Background:**

In 1980 our group set up a control program in endemic regions with CE in rural sections of Rio Negro, Argentina. Since 1997, we have used abdominopelvic ultrasound (US) as a screening method of CE in school children and determined an algorithm of treatment.

**Objectives:**

To describe the training system of general practitioners in early diagnosis and treatment of CE and to evaluate the impact of the implementation of the field program.

**Materials and Methods:**

In 2000, to overcome the shortage of radiologists in the area, we set up a short training course on Focused Assessment with Sonography for Echinococcosis (FASE) for general practitioners with no previous experience with US. After the course, the trainees were able to carry out autonomous ultrasound surveys under the supervision of the course faculty. From 2000 to 2008, trainees carried out 22,793 ultrasound scans in children from 6 to 14 years of age, and diagnosed 87 (0.4%) new cases of CE. Forty-nine (56.4%) were treated with albendazole, 29 (33.3%) were monitored expectantly and 9 (10.3%) were treated with surgery.

**Discussion:**

The introduction of a FASE course for general practitioners allowed for the screening of CE in a large population of individuals in remote endemic areas with persistent levels of transmission, thus overcoming the barrier of the great distance from tertiary care facilities. The ability of local practitioners to screen for CE using US saved the local residents costly travel time and missed work and proved to be an efficacious and least expensive intervention tool for both the community and health care system.

## Author Summary

Cystic echinococcosis (CE) is an important and widespread disease that affects sheep, cattle, and humans living in areas where sheep and cattle are raised. CE is highly endemic in rural sections of Rio Negro, Argentina, where our group is based. However, it requires continuous monitoring of both populations with human disease best assessed by means of ultrasound (US) screening. This is challenging in remote rural areas due to the shortage of imaging specialists. To overcome this hurdle, we set up a two-day training program of Focused Assessment with Sonography for Echinococcosis (FASE) on CE for family medicine practitioners with no previous experience in US. After the course, they were equipped with portable US scanners and dispatched to remote rural areas in Rio Negro where they screened patients, located and staged the cysts and decided on the treatment with the help of surgeons and radiologists in local tertiary care centers.

The need to travel to referral hospitals for traditional surgical interventions was therefore limited to a few cases. US was instead brought to rural areas thereby extending affordable healthcare to people who would otherwise not have access to it.

## Introduction

Hydatidosis or cystic echinococcosis (CE) is a disease caused by the larval stage of the cestode *Echinococcus granulosus*. It is endemic in sheep raising areas and is among the most neglected diseases in the world today [1], [2]. Despite a global burden calculated at 1 009 662 disability adjusted life years (DALYs), CE continues to be excluded from funding associated with conditions related to low socioeconomic status [3].

The effects of this neglect have a stronger impact in remote rural areas whose inhabitants have no chances of being diagnosed and treated properly without leaving their jobs and travelling long distances, sometimes taking days to reach the closest referral center. Rio Negro, situated on the northern border of Patagonia in the south of Argentina, is one such place.

The main endemic area for CE measures 143,048 km^2^ with a population at risk (e.g. shepherds, people in contact with stray dogs, etc) of 85,509 people [4], [5], [6].

**Figure 1:**
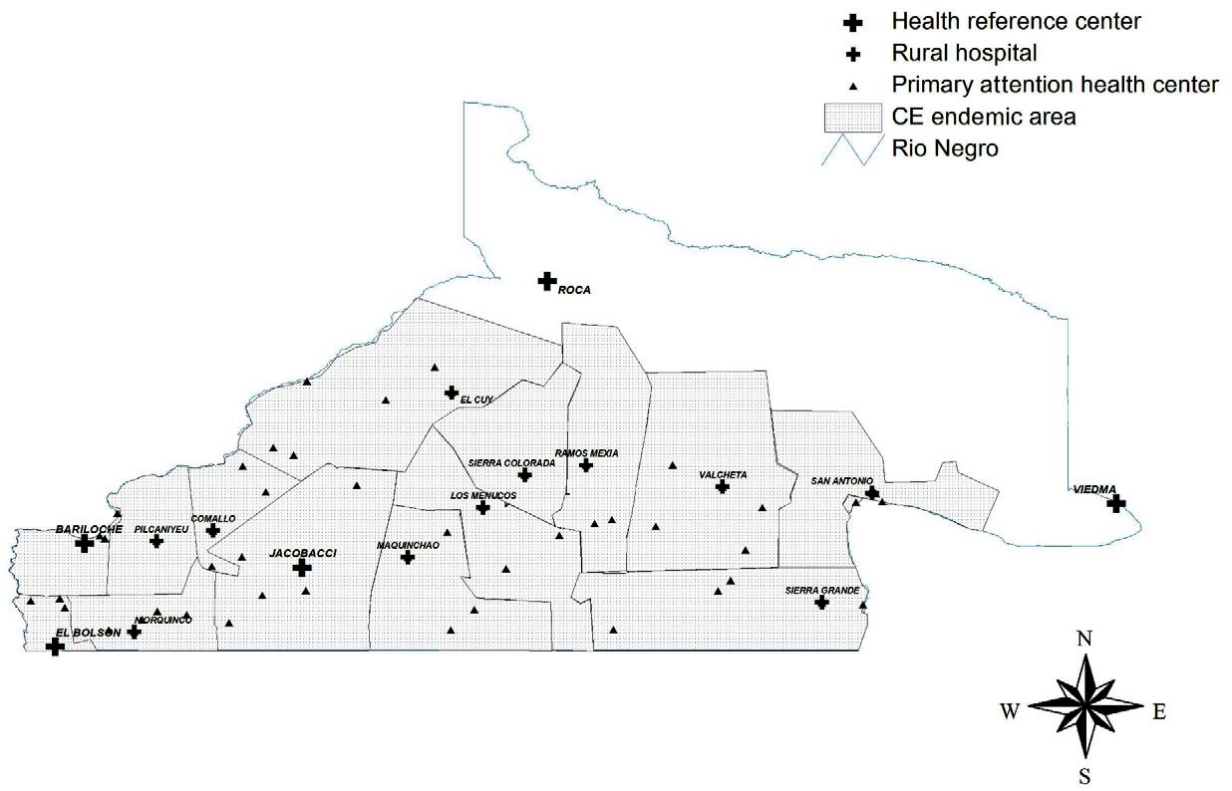
Health services of Rio Negro Province, Argentina.

The health structure in the endemic area is comprised of three tertiary care hospitals (Roca, Viedma, Bariloche), 10 rural (secondary care) hospitals and a network of 57 rural or suburban health outposts (primary care). The entire health system is staffed by nurses or other paramedics/clinical officers. Distances between a health outpost and its referral rural hospital can be as long as 120 Km by rural roads, often made impassable by the harsh weather conditions of Patagonia (ice, snow and rain in winter). Likewise, distances between rural hospitals and the tertiary care hospitals can be as long as 300 Km. making diagnosis, treatment and control of CE extremely difficult (Figure 1).

To address these problems, in 1997 our group set up a program to conduct annual ultrasound examinations of groups at risk including shepherds and children living in endemic areas. We treated active cysts with albendazole (ABZ) and monitored the response to treatment using a portable US scanner that was transported by bus to the various rural sites. The goal was to provide a cost-effective way to deliver care for CE in patients living in remote rural areas.

The sensitivity and specificity of US of abdominal CE in rural settings compared to plain radiography or computed tomography (CT) has been assessed in a previous work. In that study, 1054 schoolchildren living in rural areas were evaluated by US. Twenty-seven cases (100%) diagnosed with abdominal CE were re-examined by specialists in a tertiary care center with US, radiography and CT scan. They confirmed the diagnosis in 24 patients while 3 cases turned out to be negative. The sensitivity of US was 100% and the specificity was 95.6% [7].

In Argentina, US surveys for CE in rural areas are generally carried out by radiologists. Since they are in short supply and mostly employed in tertiary centers, the number of US exams that could be conducted in primary care settings was limited. To address this discrepancy, we set up a two-day FASE course in 2000 for general medicine physicians without previous experience in US. At the end of the course, the physicians learned to diagnose abdominal CE, treat positive cases with ABZ according to a specific algorithm and monitor the response to therapy with US under specialist supervision.

The aim of this study is to describe the training system of general practitioners in the diagnosis and treatment of CE and to evaluate the impact of the implementation of the field program.

## Materials and Methods

From 2000 to 2008, we organized an annual two-day training course in Focused Assessment with Sonography for Echinococcosis (FASE). This course was targeted to general medicine residents and general practitioners working in hospitals in rural endemic areas. Veterinarians participating in the control program also attended the course as external observers and taught CE epidemiology. The 20-hour course was limited to 20 students and included both lectures and practice with three ultrasound machines located in three consulting rooms in the “Rogelio Cortizo” hospital of Ingeniero Jacobacci. Hands-on training included scanning 200 healthy school children and approximately 30 cases of CE of different types.

The entire abdominal cavity was scanned in four basic planes as described by Saint Martin et al. [8]: transverse, sagittal, oblique subcostal and coronal supracostal. US is a very accurate imaging modality to explore solid organs in the abdomen.

All cystic masses were considered “cystic echinococcosis” until proven otherwise and further examined by the supervisors for confirmation.

The topics during the course included epidemiology, control and surveillance strategies of echinococcosis, ultrasound diagnosis of CE, including cyst classification (Gharbi and from 2007 on, WHO-IWGE), [9], [10] differential diagnosis, use of ultrasound in primary health care, organization of ultrasound surveys for CE (epidemiological objectives and evaluation of results), expectant management (“watch and wait”) for selected asymptomatic carriers, antiparasitic treatment with albendazole, surgical treatment options for CE (open or laparoscopic surgery, or minimally invasive treatments such as PAIR), provincial guidelines for diagnosis and treatment, and control activities applicable in an interdisciplinary way at secondary care centers. The course ended with a discussion about the use of ultrasound in the control program and a written exam.

The faculty included the chairs of the surgical departments, radiologists from the referral hospitals and veterinarians teaching epidemiology and control of CE. Surgeons and radiologists also acted as supervisors for the general practitioners at the rural hospitals. The screening and monitoring program was carried out annually in school children between 6 and 14 years of age in areas endemic for CE with a portable ultrasound machine. Each year the aim was to study all schools in each geographic area. The trainer, with the support of personnel from the control program, prepared the US screening schedule ahead of time including the transfer of the US scanner to all rural schools prior to arriving in the area. The schedule included screening all children along with controls of each previously diagnosed case. In addition, other groups at risk were studied, including people in the same household as diagnosed cases and sheep owners. Suspected cases of CE were sent to referral hospitals to be further investigated by specialists and the US scanner was sent by bus to the next rural hospital.

The algorithm for treatment according to cyst stage (WHO IWGE classification) and size is based on the “Clinical Guidelines for Diagnosis, Treatment of Human Hydatidosis” [11], [12], [13] updated by Resolution 3541/09 of the Ministry of Health of Rio Negro Province. Treatments were chosen by the general practitioners in the rural hospital, after consultation with the Heads of Surgery in the tertiary care hospitals and only the cases with a clear indication to surgery were referred to the surgeons.

Albendazole (ABZ) tablets were supplied at cost by the laboratory accredited by the Health Ministry of the Province of Rio Negro.

After the diagnosis, cases were geographically mapped with GPS to locate those in remote rural areas that required further monitoring and follow-up. Monitoring of treatment was carried out by the local health network (30 days after diagnosis or start of treatment, 60 days and thereafter every 3 months until completion of the first year and re-scanning patients once a year up to 10 years afterwards as indicated in the Guidelines) [12]–[13].

All the results from this paper are based on the WHO IWGE classification. We included a subdivision of type I based on the size of cyst.

### Ethics statement

Childhood screening is done as a part of the school health program through the Ministry of Health for Rio Negro. Each child's parents gave written consent prior to be scanned. All participants in the ultrasound training course gave written consent.

The Medical Committee of Control Program Against Hydatidosis in Rio Negro (established by Decree 6412/06 of the Ministry of Health of the Province of Rio Negro) gave consent for the study.

## Results

Between 2000 and 2008, 180 general practitioners and general medicine residents attended the FASE course and passed the exam.

On the first screening performed by trainees immediately after the course, all suspected cases turned out to be false positives (for specificity) when the tutors (radiologists) rescanned the patients.

The cost of having a specialist rescanning the patients to look for diagnostic errors was, however, lower to that of false negatives with ensuing complications due to an undiagnosed cyst.

From 2001 to 2008 the trainees performed 22,793 ultrasound studies on children between 6 and 14 years of age and diagnosed CE in 87 cases (0.4%) with an average of 8.9 years with 94 cysts identified (85.2% CE1 or CE2 and 6.4% CE4 or CE5). Forty-nine (56.4%) cases were enrolled in the ABZ protocol, 29 (33.3%) were enrolled the expectant management (watch and wait) protocol and 9 (10.3%) were treated with surgery (Table 1).

**Table 1:** Number, type and localization in CE carriers.

Number, size and stage of cysts		Cases diagnosed 2001–2008	Initial treatment of cases	Control June 2010 treatment, cyst stage
Average years of age		8.9		12.9
Diagnosed with CE		87 (0.4)		77
N° cysts		94 (1.2/patient)		67
	CE1<3 cm	22 (21.6%)	22 cases WW	14 (18.2%) CE1
	CE1 3–10 cm	59 (57.8%)	38 cases ABZ, 6 SURG	
	CE2/CE3b	5 (4.9%)	3 cases ABZ, 2 SURG	1 (1.3%) CE2
Size and Stage of cysts	CE3a	10 (9.8%)	1 case WW, 8 ABZ, 1 SURG	10 (13.0%) CE3
	CE4	5 (4.9%)	5 cases WW	22 (28.6%) CE4
	CE5	1 (1.0%)	1 case WW	15 (19.5%) CE5
	NO CYST			15 (19.5%)
Site	Liver	91 (96.8%)		
	Kidney	3 (3.2%)		

Ultrasound surveys in children, Rio Negro, Argentina.

Footnotes:

WW: Watch and wait. ABZ: Albendazole. SURG: Surgery.

World Health Organization standardized classification of ultrasound pattern of cystic Echinococcosis (CE). CE1: unilocular cyst. CE2: multivesicular cyst. CE3a: detached membrane. CE3b multivesicular cyst with solid component. CE4: heterogenous pattern. CE5: calcified.

Note: 1 patient with 2 cysts may have 1 cyst <3 cm and 1>3 cm. The treatment listed by patient not by cyst. The same happens when a patient has 1 CE1 and 1 CE4 : the treatment is aimed at the former.

Of the 22,793 ultrasounds performed, some may have been inadvertently repeated on healthy children despite being negative and this might explain the low prevalence of 0.4%.

The last control used for our analysis occurred in 2009. The average length of follow-up was 12.9 years for each patient. Of those scanned, 15 (24.2%) cysts remained CE1 or CE2, with their size unchanged and 37 (59.7%) were CE4 and CE5. Cysts disappeared in 6 (8.7%) cases (Table 1).

By 2009, follow-up by general practitioners of cases first diagnosed in 1998–1999 reached 100% of cases at 5 years and 64% at 10 years from diagnosis [11], [12]. Follow-up of patients first diagnosed in 2000–2008 reached 88.5% of cases by June 2010.

A detailed description of the evolution of diagnosed cases has been discussed in previous reports [12], [13].

## Discussion

Control programs for CE have been started in various endemic areas throughout the world. [14]. In the Province of Rio Negro it was started in 1980, based on the deparasitization of dogs and epidemiological surveillance using serology from 1980–1997 and subsequently abdominopelvic US scans. Positive cases were subsequently studied with the imaging techniques available at the time (X-Rays, scintigraphy, CT and US), and surgery was performed after determining the cyst location [5], [6].

In 1997, US was adopted as the method of choice for screening of CE due to its greater specificity and sensitivity compared with serologic tests [7], [15], [16], [17].

Meanwhile, new treatment options had been made available with the introduction of benzimidazoles and of percutaneous treatments [18]. As the limitations of surgery (morbidity, mortality, relapses depending on type of surgery and available medical facilities) became apparent compared to new therapeutic options [19], the US classifications of CE became a key element in clinical decision making and a more rational allocation of patients to treatment was implemented. For hepatic CE, this is based on cyst stage (active, transitional, inactive, complicated or no complicated) and size [20], [21], [22], [23].

Experience has shown that small, active and uncomplicated cysts tend to respond well to ABZ, thus making surgery unnecessary [24].

Field studies in the Rio Negro Province showed that treatment of active cysts in young asymptomatic carriers with ABZ, in 56% to 88% of patients, resulted in a partial or total involution, the latter being a complete solidification, or stages CE4 and CE5 [12], [13].

Due to the lack of ionizing radiation, US is eminently repeatable and therefore the best tool to monitor response to treatment, which in CE is a long term endeavor [18]. The cost of ABZ treatment (for 4 months) is far lower than that of surgery in Rio Negro, being estimated at around 1,350 USD against 4,596–5,936 USD for surgery [25].

Medical treatment of CE has reduced the need for hospitalization, the general healthcare costs and social cost by reducing the number of work days lost not only from treatment but also the time necessary for rural inhabitants to travel to tertiary care centers [25].

Moreover, recent clinical observations have confirmed findings from our previous studies that while selected cyst stages responded favorably to medical treatment, others needed no treatment at all [1], [2], [22], a finding whose consequences include the lowering of health and social costs of CE.

On the epidemiological side, US allows for the assessment of the effects of control programs on the human population of the targeted areas by evaluating cyst size and stage [13], [26]. With successful control programs, fewer active small cysts are expected to be seen in the population surveyed over time [14].

US is no longer limited to radiologists and is now being utilized by many different specialties [27]. As an example, in Emergency Medicine, FAST (Focused Assessment with Sonography for Trauma) can be quickly taught to surgeons and emergency medicine specialists and has revolutionized the approach to blunt abdominal trauma and other acute surgical conditions [28], [29].

Although the clinical management of many infectious diseases benefits from clinical US [30], this tool is especially useful in CE, where its use spans epidemiology, diagnosis and staging, guide for percutaneous treatments and follow-up. Despite this, it has rarely [7] been used for active case finding, clinical decision-making and follow-up in rural areas. This is currently changing thanks to the increasing use of portable scanners [31] and by teaching non-radiologists a limited set of specific skills under the supervision of experienced specialists.

This idea has been used in other diseases, for example extrapulmonary tuberculosis in rural areas of South Africa where HIV is highly endemic [32] and will be further extended with the continuing miniaturization of scanners [33].

While long term programs are needed for a more comprehensive on-site training [34], [35] in low resource settings, short focused US courses on specific conditions should also be developed to address the lack of specialists in remote areas.

With this experience, the training of general practitioners allows for an annual mass screening program by US and the allocation of patients to treatment with ABZ. Although all the trainees who participated in the screening for CE with US have left due to the high turnover of rural doctors (moving to tertiary care hospitals) we have found that having a short ultrasound teaching course allows for a smooth transition and ongoing continuing medical education of new physicians. We believe that this approach deserves to be extended to other rural areas in countries where CE is endemic. US is a wonderful technology that can be applied to the diagnosis and treatment of CE in areas with only rural hospitals and schools, including the use of health care workers with no previous experience in ultrasound in the first step of the diagnosis [8], [36].

Portable US scanners and a short FASE course for general practitioners, together with the collaboration of veterinarians, surgeons, radiologists and paramedics and the availability of ABZ, helped prevent clinical problems resulting from unchecked cyst growth, minimized social and health care costs and avoided expenses related to time spent by rural patients travelling long distances to tertiary care centers.

The course provided the local population the benefit of having US performed at no cost and with minimal disruption to their lives given the lack of transportation time to referral hospitals or clinics.

Timely diagnosis and early treatment produced a marked decrease in morbidity, number of hospital admissions and the strain on medical services in patients with CE [4], [11]. The need to travel to referral hospitals for the traditional surgical interventions was thus limited to a few cases and ultrasound was brought to rural areas instead, extending affordable healthcare to people who would otherwise not have access to it.

Just as US revolutionized the clinical management and control of CE, focused US training has the potential to extend its benefits to underserved populations at an affordable cost for the community.
